# Focusing on optimality for the translation of precision medicine

**DOI:** 10.1017/cts.2022.438

**Published:** 2022-08-04

**Authors:** Anna R. Kahkoska, Kristen Hassmiller Lich, Michael R. Kosorok

**Affiliations:** 1 Department of Nutrition, University of North Carolina at Chapel Hill, Chapel Hill, NC, USA; 2 Department of Health Policy and Management, University of North Carolina at Chapel Hill, Chapel Hill, NC, USA; 3 Department of Biostatistics, University of North Carolina at Chapel Hill, Chapel Hill, NC, USA; 4 Department of Statistics and Operations Research, University of North Carolina at Chapel Hill, Chapel Hill, NC, USA

**Keywords:** Precision medicine, optimality, health outcomes, systems science

The scientific field of precision medicine aims to generate evidence to deliver the right treatment to the right patient at the right time, relying on individual-level data and statistical modeling methods to offer insights into more precise risk stratification, prediction, and treatment recommendations [1–3]. Early success stories have sparked excitement [4–6]. Fueled by growing momentum, the assumption that precision confers optimality has become so implicit in conversations surrounding precision medicine that it is typically not made explicit – the promise of optimization is just assumed to be part of how precision recommendations are made. Often, this assumption is upheld, and “precision medicine” solutions to clinical challenges do confer improvements over their “one-size-fits-all” counterparts. The utility of precision medicine is particularly evident in heterogeneous populations [1], and the quality of insights will likely only increase as richer data from -omics, electronic health records, wearables, and environmental databases are incorporated into precision medicine models of complex disease processes and treatment mechanisms [6,7].

Yet, while entangled, precision and optimality are fundamentally different. In the context of precision medicine, to be precise is to be specific or “tailored” to an individual patient, or a subgroup of patients, rather than reflective of an entire population. To be optimal is to be most favorable or desirable. While multiple approaches to statistical modeling or clinical care may increase precision, optimality implies an underlying and direct comparison to establish the best result obtainable, under specific conditions, to maximize or minimize one or more specific outcomes.

The concept of optimality, or the task of optimization, can be found across many disciplines, including those in science, mathematics, and engineering; it is also ubiquitous in business, finance, and policy, among other areas [8,9]. Many pared-down optimization problems share several key features: objectives (i.e., what should be maximized or minimized), decision variables (i.e., what can be modified to achieve optimization), and constraints (i.e., limits to define what is and is not feasible) [9]. Our goal herein is not to provide an exhaustive review of these definitions. Instead, we aim to formally introduce the need for “recentering” the concept of optimality within precision medicine, illuminating where and why an explicit focus on optimality is needed to augment the existing emphasis on precision. Our thesis is that precision without intentional and sufficiently broad optimality is, in short, doomed to fail when it comes to translating precision medicine to patient care.

To date, the field of precision medicine has generated tremendous insights into mechanisms and models of individualized disease processes and treatment responses via both existing and new data sources, laboratory techniques, and analytic approaches. Yet, as the field advances, *mechanisms and models* will need to be translated to *tools and interventions*. Though they are entirely sufficient for individual steps of the discovery pipeline, it is likely that existing indices of clinical effects and statistical rigor construct a view of optimality that will not sustain precision medicine as a treatment paradigm.

Consider a hypothetical scenario in which there is a new treatment for a common disease. Compared to the standard of care, and among a subset of patients, the treatment confers a greater clinical benefit, but it is also more expensive and associated with a different profile of off-target effects; this is a promising setup for precision medicine analytics to guide the delivery of the new treatment to the patients who may benefit, ideally as part of routine clinical care. Though there may be multiple scientific approaches that shed insight on heterogeneity in patient characteristics, treatment responses, and side effects, those which lack rigorous data on biomarker–treatment interactions may be illuminating in the discovery pipeline, but not actionable, thus offering no clear path to optimizing patient outcomes. Alternatively, even if new evidence is actionable, approaches that focus on clinical benefits but ignore costs to patient or providers may be non-starters in real, complex, care settings; introducing precision within routine care often carries a cost (e.g., tool development, patient and provider education, time, resources, etc.) [2,10], and failure to consider what is *optimal for the spectrum of stakeholders including providers and healthcare systems*, and *feasible given system constraints and limited resources*, may limit the potential impact of an intervention due to implementation and dissemination challenges. Finally, and most importantly, a movement toward precision medicine that fails to consider what is *optimal for populations* carries a grave risk to propagate and augment the effects of structural racism and other health inequities, particularly with regard to the social determinants of health, access to care, and the affordability of individualized regimens [11,12].

A further challenge is rooted in the fact that actionable precision medicine science will be translated to patient care not as one treatment or intervention but rather as a *suite* of treatments or interventions. Put otherwise, precision medicine– as a treatment paradigm– offers an overarching framework for care delivery that selects one intervention, from multiple possible interventions, to account for individual or subgroup variability in genetic, phenotypic, lifestyle, and environmental factors that may impact the effectiveness of selected interventions. Both development and evaluation of precision medicine in care thus requires a wider view to reflect the complexity matching multiple potential interventions to individual patients, capturing short- and long-term impacts at the patient, provider, healthcare-system, and population levels. In sum, optimality, in precision medicine, is multifaceted, dynamically complex, and context specific; these features are outlined in Table 1.

**Table 1.** Features of optimality for precision medicine as a treatment paradigm

Feature	Definition	Explanation	Recommendations for translation
***Optimality is multifaceted***	Multifaceted implies the existence of many (i.e., two or more) aspects that are relevant for evaluation	Compared to singular, discrete interventions with a primary outcome of interest, there may be multiple, diverse effects that are relevant as “outcomes” of a precision medicine model for care delivery, owing in part to the large number of scientific, clinical, administrative, and patient stakeholders	Transitioning from discovery science to precision medicine-based clinical care may necessitate integration of mechanistic signals with patient- and provider-oriented outcomes as key evaluation measures. Patient preferences may be particularly salient for long-term interventions or those with one or more behavioral components, in addition to decision alternatives that integrate patient preferences, capabilities and constraints. Impacts on clinical workflow, resource efficiency (particularly where resources are limited), and cost-effectiveness for patients and care or community systems must also be considered.
***Optimality is dynamically complex***	Dynamic complexity refers to when outcomes cannot be explained through simple cause and effect relationships and instead represent influences from many related factors; the interactions between factors may be nonlinear, have time delays, and contain feedback loops that reinforce or balance changes within a web of interacting factors [23]	Routine care settings offer a “messy” environment that often functions as part of one or more complex systems. A system is defined as a web of multiple, heterogeneous, and interconnected components that together produce emergent effects that cannot be intuited from understanding the individual components in isolation [18,24]. Dynamic complexity within care systems resulting from the hidden interactions between known and unknown factors may make it difficult to predict or understand when and why precision medicine may be more or less optimal than the standard of care. Dynamic complexity may also result in different short-term and long-term effects of precision medicine care delivery models and the interventions they comprise.	Evaluation processes must account for interaction among often tightly coupled and interdependent phenomena, as well as the role of feedback from decisions, misaligned incentives, and changing information. Interdisciplinary teams are necessary to predict, interpret, and integrate changes to healthcare resources, best practices, and communities over time, facilitating reverse translation through the bidirectional flow of knowledge and innovation between real-world needs, clinical practice, and evidence generation
***Optimality is context specific***	Context is the setting in which an intervention is implemented, which may refer to a physical or virtual location, a clinical circumstance, and the characteristics of a patient population and the barriers/facilitators to care including socio-cultural factors	The physical and virtual systems in which precision medicine will be deployed ultimately shape the barriers and constraints of individualizing care. The ultimate cost associated with precision medicine interventions and the benefits are both determined and absorbed by local systems. Further, in addition to differences in demographic, biologic, and clinical factors, individuals also bring a rich social and cultural context to their care; translating precision medicine across populations will require tailoring of care models to embrace diversity in the factors that impact on health and wellness outside of the clinic walls.	Quantitative and qualitative data collection, diverse stakeholder engagement, and linkages of existing data from care and community systems are needed to yield individual-level data and system-level data for both planning and evaluating precision medicine-based care models in new settings and contexts. Patient and community stakeholders should be engaged to guide processes to collect and use individual-level data for more precise care recommendations that is culturally sensitive, equitable, and engenders public trust.

Despite the daunting nature, defining the intersection of precision and optimality is critical for the later stages of translating precision medicine science into practice [4,13]. The precision framework introduces a degree of complexity at the systems level, but the counterbalance of optimality may help to identify and prioritize opportunities where precision approaches are simple yet effective, including those which rely on clinically accessible data for tailoring and can be operationalized as simple, parsimonious decision rules. Further, an unrelenting focus on precision at the level of individual differences and effects, taken out of context from routine care systems and their constraints, may fail to recognize subpopulations for whom tailoring yields clear benefits that outweigh the costs and resources needed for tailoring. Refocusing optimality alongside precision with a wider angle view may help to inform whether, when, and how to increase precision while balancing limited resources, enhancing *translation to practice* by generating strategies to yield a global value that exceeds the costs of local implementation. Finally, with a proper framework to evaluate the tradeoffs between the health of populations and the health of individuals, there lies a path for the ultimate translation of precision medicine *to communities*: improved population outcomes conferred by many smaller, individual-level improvements [1].

We propose that several existing scientific methodologies can help to illuminate aspects of optimality in the context of precision medicine as a treatment paradigm. Implementation science houses theories to understand barriers and facilitators to changing practice, as well as scientific methods to promote and optimize precision medicine interventions given the unique constraints and priorities of different, local healthcare and community settings [2]. The field of systems science complements implementation science methods by offering further, complexity-aware approaches for optimizing of precision medicine in real-world systems in which new models of care will be deployed and sustained [14–17]. Common techniques include mapping stakeholders’ understanding of system structure, patient experiences, and health services (e.g., system dynamics or process flow diagraming) and simulation modeling to support decision making surrounding the delivery of interventions (e.g., cost-effectiveness modeling, agent-based modeling, and discrete event simulation modeling/queueing) [18]. Finally, advancements in biostatistics have yielded machine learning-based methods to directly estimate optimal interventions for individual patients, picked from a set of potential interventions, to improve one outcome or set of outcomes, based on an individual’s demographics, clinical status, or response to past treatment(s)[1]. Critically, these algorithms can also be used to optimize the effect of treatments or treatment sequences on long-term rather than proximal outcomes [1]. There is also a growing focus on developing simple and interpretable artificial intelligence algorithms for precision medicine [19] and evaluating how different sets of biomarkers can be combined to yield optimized treatment recommendations, yielding new opportunities to refine algorithms for different settings where variable patient data may be readily available [20,21].

At the same time, optimality is an inherently humanistic concept; particularly when the optimization problem involves clinical medicine and broader health promotion. Learning what is optimal for precision medicine will require a careful mix of objective and subjective appraisal, as well as the reconciliation of the different ideas, values, and constraints across different stakeholders of care delivery and population health. Despite the call for development of interdisciplinary team-based initiatives [2,22], guidance on the assembly of such cross-disciplinary teams is lacking. The need for multifaceted, dynamically complex, and context-specific evaluation could provide the framework to guide inclusion of the right stakeholders at the right time to achieve effective and equitable translation.

In many ways, the need to re-center optimality alongside precision reflects the readiness of the scientific field to make larger translational steps than before. The research questions have matured from “proof-of-concept” (i.e., how to characterize heterogeneity in phenotypes, prognosis, and treatment responses) to those focused on how to leverage those insights as part of a more precise model of healthcare delivery that balances potential improvements in individual-level outcomes (e.g., clinical metrics and patient preferences) with the outcomes that are important to other stakeholders, the reality of clinical workflows and limited system resources, a need for cost-effectiveness, and an accountability to improving health equity and population health. Fundamental questions that must be addressed for effective translation of the growing evidence from precision medicine science: What is optimal, from whose perspective, over what time frame, and in what setting? Making explicit where the union of precision and optimality lies, for whom and in what context, is critical to driving precision medicine’s long-awaited translation to clinical and community practice.
